# Management of Hypertension in Diabetic Kidney Disease

**DOI:** 10.3390/jcm12216868

**Published:** 2023-10-31

**Authors:** Gates B. Colbert, Mohamed E. Elrggal, Anna Gaddy, Hector M. Madariaga, Edgar V. Lerma

**Affiliations:** 1 Division of Nephrology, Texas A&M University College of Medicine at Dallas, Dallas, TX 75246, USA; 2 Kidney and Urology Center, Alexandria 21922, Egypt; 3 Division of Nephrology, Medical College of Wisconsin, Milwaukee, WI 53226, USA; 4 Lahey Hospital & Medical Center, Burlington, MA 01805, USA; 5 Section of Nephrology, Department of Medicine, University of Illinois at Chicago, Chicago, IL 60612, USA; nephron0@gmail.com

**Keywords:** hypertension, RAASi, aldosterone, SGTL2i, mineralocorticoid receptor antagonist

## Abstract

Hypertension is a critical component of cardiovascular disease progression in patients with chronic kidney disease, and specifically diabetic kidney disease (DKD). Causation versus correlation remains up for debate, but what has been confirmed is the delay of DKD progression when hypertension is controlled or moved to guideline drive ranges. Many medications have been studied and used in real world experience for best outcomes, and we discuss below the proven winners thus far making up the renin angiotensin aldosterone system. As well, we discuss guideline changing medications including sodium-glucose cotransporter 2 inhibitors and newer generation mineralocorticoid receptor antagonists. With the growing prevalence of diabetes and DKD in the population, newer agents are emerging in multiple drug class and will be highlighted below. Clinicians continue to search for the optimal care plans for this challenging patient population.

## 1. Introduction

**Figure 1:**
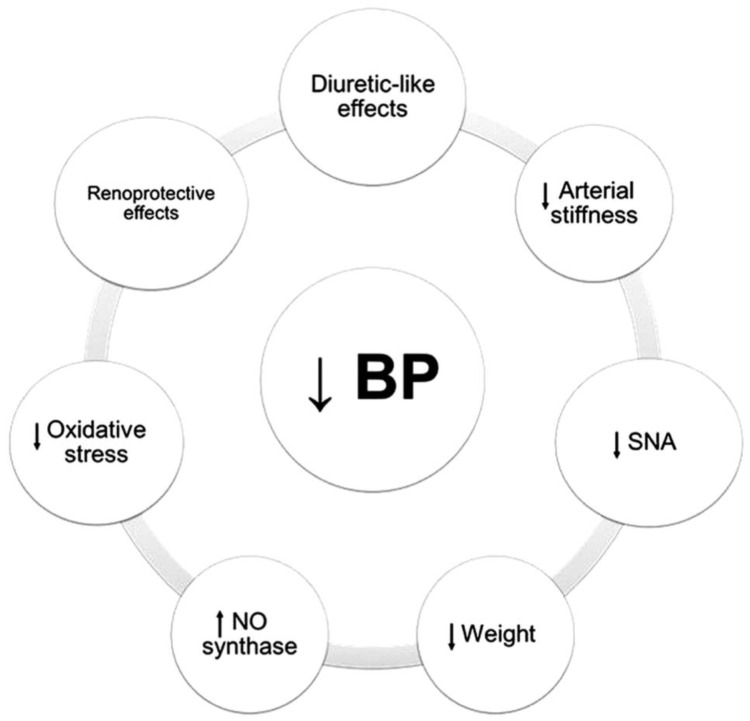
Pathophysiological mechanisms of lowering blood pressure. Abbreviations: BP, blood pressure; NO. nitric oxide; SNA, sympathetic nervous. Source: Sanidas et al. [4].

When considering the treatment of hypertension in diabetic patients with chronic kidney disease, two main questions arise: What should our target blood pressure be, and what pharmacologic interventions should we use to achieve these targets? The answer to the first question has been the topic of much debate over the past decades, with a shift from a less intensive 130/80 goal historically to a trend toward tighter control post-SPRINT reflected in 2021 KDIGO Guidelines [1]. Post-hoc analysis of the IDNT [2] and RENAAL [3] show a strong association between tighter systolic blood pressure control and decreased progression of chronic kidney disease. In fact, post-hoc analysis of RENAAL reported that a 10-mm Hg rise in baseline SBP increased the risk for end-stage kidney disease (ESKD) or death by 6.7%. However, professional societies do not agree on a specific systolic goal, given the potential harm of hypotension when targeting very low blood pressure. As such, a pragmatic approach is required to personalize systolic blood pressure goals as tolerated but maximize benefit by using the correct tools. As clinicians, we have a vast array of anti-hypertensive medications at our disposal but should focus on those that provide additional benefit to our diabetic patients beyond targeting a systolic goal, whatever that goal may be. As we must consider the mechanistic targets that need modification from a pathologic sequence as reviewed in Figure 1.

## 2. Angiotensin Converting Enzyme Inhibitors and Angiotensin-Receptor Blockers 

Pharmacological blockade of the renin–angiotensin–aldosterone system (RAAS) using angiotensin-converting enzyme inhibitors (ACEi) and angiotensin receptor blockers (ARB) has become the cornerstone for patients with type 2 diabetes (T2D) and kidney involvement. Several clinical trials have demonstrated that the use of RAAS inhibitors (RAASi) slows chronic kidney disease (CKD) progression and reduces proteinuria in patients with proteinuric CKD, including diabetic kidney disease (DKD) [5,6,7,8,9,10]. 

There are two seminal randomized clinical trials showing kidney benefits from RAASi independent of their blood pressure-controlling effects: the IDNT [2] and the RENAAL [3] trials. The trials compared the use of ARB versus placebo or ARB versus calcium channel blockers (CCB) or placebo in a double-blinded design. RENAAL included patients with and without hypertension, while IDNT excluded those with normal BP. Both trials included patients with kidney impairment (IDNT: baseline serum creatinine of 1.67 mg/dL, RENAAL: baseline serum creatinine 1.9 g/dL) and severe albuminuria (IDNT: 2.9 g/d proteinuria, RENAAL: albumin–creatinine ratio 1237 mg/g). Irbesartan in the IDNT study resulted in a 20% (RR:0.80; 95% CI: 0.66–0.97) risk reduction in the primary composite endpoint (doubling of serum creatinine, ESKD, death from any cause) versus placebo and a 23% reduction versus amlodipine (RR:0.77; 95% CI: 0.63–0.93); while losartan in the RENAAL study caused a 16% (RR:0.84; 95% CI: 0.72–0.98) reduction in the composite outcome of death, dialysis, and doubling of serum creatinine compared to placebo. Unfortunately, in RENAAL, as part of the secondary outcomes, there was no mortality benefit (21% vs. 20.3% (*p* = 0.88), but overall, the trial has been a cornerstone for establishing RAASi as a major difference makers.

On the other hand, for patients with CKD, hypertension, diabetes, and no albuminuria, current evidence does not support clear clinical benefits of RAASi for CKD progression, and other antihypertensive drugs are appropriate for BP management; however, the Kidney Disease Improving Global Outcomes guidelines (KDIGO) stated that these patients may be preferably treated with RAASi (ACEi or ARB), given the cardiovascular (CV) protection benefits, especially in patients with a higher glomerular filtration rate (GFR)1. There has been some criticism about the superiority of RAS inhibitors compared to other hypertensive agents in albuminuric patients [11], but generally, the presence of protein should be considered an indication for the use of ACEi or ARB. 

It is also important to consider that beyond the need for renoprotection, patients with kidney insufficiency have a substantially increased risk of cardiovascular death [12]. The HOPE study reported cardiovascular benefits with an ACEi versus placebo in people with diabetes at high CV risk, even those without a diagnosis of hypertension, and independent of the BP control effect [13]. In a pre-specified subgroup analysis, patients with CKD (1/3 had diabetes) with mild or no albuminuria, ACEi use reduced the incidence of the primary outcome (incidence of cardiovascular death (reduced by 20%), myocardial infarction (reduced by 26%), or stroke (reduced by 31%)) during a mean follow-up duration of 4.5 years. 

### 2.1. Selection of a RAS Inhibition Agent and Dose

Overall, there was no difference between ACEi and ARB for the outcomes of MI, stroke, and kidney function in CKD patients with diabetes, with or without albuminuria [9]. However, a recent meta-analysis including 119 trials found that survival benefits were reported for ACEi but not for ARB in patients with CKD and DKD [14,15]. Any combination of ACEi and ARB or direct renin inhibitors is strongly discouraged as it has been associated with hyperkalemia and hypotension without any additional benefit [16,17].

RAASi agents should be utilized using the highest approved tolerated dose because the proven benefits were achieved in trials using these doses. Changes in BP, serum creatinine [18], and serum potassium [19] are expected after the initiation or increase in the dose of a RAASi; therefore, a regular check of BP, serum creatinine, and potassium should be performed within 2–4 weeks of any change. The strategy of gradual up-titration with careful blood pressure and symptom monitoring is prudent. 

### 2.2. Other Considerations: When to Stop RAS Inhibition

Given the potent morbidity and mortality benefits described in patients with DKD, every effort should be made to continue therapy with RAS blockade. In fact, discontinuation of RAS blockade for hyperkalemia was associated with increased mortality and CV events [19]. RAASi should be continued unless serum creatinine increases by more than 30% within 4 weeks of initiating RAASi or increasing its dose. Data from the RENAAL study found that patients who had already doubled their serum creatinine levels during the study and remained on RAASi therapy had a significantly delayed onset of dialysis by a mean of 6 months. Current guidelines reinforce the recommendation to continue RAASi agents as long as possible for the most CV benefits. New oral potassium binders (patiromer and sodium zirconium cyclosilicate) can be a useful tool in patients confronted with mild hyperkalemia and no electrocardiogram abnormalities [20,21]. 

Traditionally, many clinicians discontinued RASi in very advanced CKD to try to preserve eGFR. The STOP-ACE trial showed that in advanced CKD, eGFR < 30 mL/min/m^2^, discontinuation of RAASi did not significantly improve kidney function compared to the RAASi continuation group and did not prevent any patients from starting dialysis during the study period [22] This supports the use of RASi continuation in advanced CKD, maximizing its anti-proteinuric and cardiovascular benefits throughout the entire CKD spectrum. If RASi cannot be used or causes side effects, alternative agents such as CCB or diuretics may be used to control hypertension. In patients with severe albuminuria, non-dihydropyridine agents (such as diltiazem and verapamil) are preferred over dihydropyridine CCB, as they can reduce albuminuria [23].

## 3. Mineralocorticoid Receptor Antagonists

Though the combined use of ACEi and ARBs is not recommended, there is certainly a role for additive blockade of the RAS system with the addition of mineralocorticoid receptor antagonists. This class of medication has been studied extensively in patients with heart failure, in whom ACEi and ARBs are the standard care.

Non-specific MRAs, such as spironolactone, eplerenone, and drospirenone are steroidal in nature. These drugs bind directly and block the mineralocorticoid receptor, preventing its activation by aldosterone or 11-deoxycorticosterone. They also inhibit the receptors stimulated by testosterone and dihydrotestosterone, leading to common side effects such as gynecomastia, breast tenderness, and feminization. Specific MRAs are non-steroidal and were designed to minimize those adverse effects, including finerenone and esaxerenone. 

As previously discussed, patients with DKD are at high risk of cardiovascular events. Though not specific for patients with diabetic kidney disease, the EPHESUS trial assessed the efficacy of eplerenone in patients with heart failure and myocardial infarction, demonstrating a benefit in decreasing mortality (RR 0.85, 95% CI 0.75–0.96 *p* = 0.008), lower risk of sudden cardiac death (RR 0.79, 95% CI 0.64–0.97% *p* = 0.03), and improvement in systolic and diastolic blood pressure compared with placebo. Notably, these benefits came with a small additional risk of hyperkalemia (5% versus 3.9% in placebo) despite the exclusion of patients with hyperkalemia at initiation [24]. The EMPHASIS-HF trial also demonstrated significant cardiovascular benefits in patients with HFrEF < 35% and NYHA class II randomized to eplerenone vs. placebo [25]. Patients were either on RAAS blockade or a beta blocker, and after a 1.8-year follow-up, the eplerenone group demonstrated a risk reduction of composite cardiovascular death or heart failure hospitalization (HR 0.63; 95% CI 0.55–0.76, *p* < 0.001) as well as an overall reduction in systolic blood pressure of 2.5 ± 17.2 mmHg in comparison to placebo (*p* = 0.001).

The later FIGARO trial confirmed these cardiac benefits in a diabetic population when comparing finerenone to placebo in diabetic CKD patients already on ACEi/ARB; the mean difference in change from baseline in systolic blood pressure was −3.5 mmHg at month 4 and −2.6 mmHg at month 24 [26]. The primary outcome, assessed in a time-to-event analysis, was a composite of death from cardiovascular causes, nonfatal myocardial infarction, nonfatal stroke, or hospitalization for heart failure. In 3.4 years, this composite occurred in 12.4% of the finerenone group compared to 14.2% of the placebo group (hazard ratio, 0.87; 95% confidence interval [CI], 0.76 to 0.98; *p* = 0.03), with the benefit driven primarily by a lower incidence of hospitalization for heart failure. Not all individual secondary endpoints were significant, but the trial was positive overall for the composite endpoint.

The benefits of MRA addition are not limited to decreased cardiac mortality. However, the addition of MRA to ACEi/ARB in proteinuric diabetic patients has long been a treatment strategy to improve albuminuria and, theoretically, slow the progression of kidney disease. Though no large clinical trials had investigated the addition of a mineralocorticoid receptor antagonist to standard RAS blockade (ACEi/ARB) in diabetic patients for renoprotection with eGFR endpoints, meta-analyses showed a significant and dramatic (near 30%) reduction in albuminuria with this combination [26].

Then, in the FIDELIO-DKD trial, patients with CKD and type 2 diabetes mellitus were randomized to finerenone or placebo [27]. In this study, finerenone demonstrated a modest change in mean systolic blood pressure (baseline to 1-month change of –3.0 mmHg) but a significant and more robust improvement in the composite outcome of kidney failure, 40% decreased eGFR, or death from renal causes. In 2.6 years, 17.8% of patients in the treatment group experienced this outcome compared to 21.2% in the placebo group. A secondary benefit was seen in the decreased cardiac events and death, which occurred in 13.0% of the treatment group versus 14.8% of the placebo group. This study provided the first evidence that mineralocorticoid receptor antagonists were effective at preventing the progression of kidney disease in diabetic patients, rather than relying on the surrogate outcome of proteinuria as in prior trials.

### Other Considerations: Preventing and Managing Hyperkalemia

One of the risks of using MRAs is the associated risk of hyperkalemia, which is theoretically increased when blocking steps of the RAAS in sequence. Indeed, a meta-analysis of small trials adding steroidal and non-steroidal MRAs to ACE/ARB found that the risk of hyperkalemia was higher with the combination of medications than with ACE/ARB alone. This was not seen in the EMPHASIS trial of heart failure patients, in which 93% of patients were on ACE/ARB concurrently with eplerenone and which notably excluded patients with a serum potassium of >5 mmol/L from randomization. Similarly, the FIDELIO-DKD trial did not randomize any patients with a starting potassium of >4.8 mmol/L and noted fairly low risk of mild (21.4%) and moderate (4.5%) hyperkalemia over 2.6 years of follow-up. At initiation, 2.5% of patients were on potassium-lowering medications at baseline, and by the end of the study, approximately 10% of patients needed such medications [28,29].

The design of the more recent studies of finerenone can be considered when making therapeutic decisions for our patients. Patients in whom serum potassium is near or above 5 mmol/L on ACE/ARB alone should be considered to be outside the safety parameters included within these studies, and the decision to start MRA should be made carefully. In all patients, routine potassium monitoring, occasional temporary treatment interruption, and dose reduction are advised in the event of hyperkalemia. Combination with oral potassium binders can also be considered for hyperkalemic patients on RAS inhibition therapy. This strategy was tested in the OPAL-HK Trial and found to be effective in patients with CKD [20]. It is worth noting that the percentage of patients in this trial who were receiving MRA or dual blockade with ACEi/ARB and MRA was relatively small, but there is no reason to suspect oral potassium binders would not be as effective in this setting, given their distinct and unrelated mechanism of action. 

## 4. Sodium-Glucose Co-Transporter 2 Inhibitors

Although thought of as a glucose-lowering medication with proven cardiovascular benefits, sodium-glucose co-transporter 2 inhibitors (SGLT2i) have shown positive effects on blood pressure. Early evidence in the EMPA-REG OUTCOME and CANVAS trials demonstrated reduced blood pressure in treatment groups, the cause of which has been the subject of debate and research [30,31].

The mechanism of this observed blood pressure-lowering effect is thought to be secondary to natriuresis and osmotic diuresis via local inhibition of RAAS. The increased sodium delivery to the juxtaglomerular apparatus is thought to cause an increase in sodium excretion by 15–20% [32]. These hemodynamic effects may provide protection against heart failure by improving filling conditions and reducing whole-body sodium content. This effect was demonstrated by Ferrannini et al. that one dose of empagliflozin given to individuals with diabetes led to mild diuresis and blood pressure reduction within one hour of treatment, in addition to a drop in creatinine clearance, an effect that persisted with chronic use [33,34]. Experimental models have demonstrated that SGLT2 inhibitors also decrease intraglomerular capillary hypertension and hyperfiltration, leading to a reduction of albuminuria and oxygen demand for tubular reabsorption [35]. Chemical denervation in mouse models of nephrogenic hypertension reduced BP and SGLT2 protein expression, suggesting that there is crosstalk between the sympathetic nervous system and SGLT2 regulation with possible effects on endothelial function as well [36,37].

In 2015, investigators evaluated the blood pressure effect of empagliflozin in patients with type 2 diabetes and hypertension, observing a systolic BP reduction of 3.4 mmHg with 10 mg of empagliflozin and 4.1 mmHg with 25 mg of empagliflozin, respectively (95% CI –4.78 to –2.09; *p* < 0.001) [38]. In a trial of dapagliflozin compared to placebo, systolic BP was reduced by –4.28 mmHg (95% CI 6.54 to –2.02; *p* = 0.0002). SGLT2i had a synergistic effect with calcium channel blockers and beta-blockers but not with thiazide diuretics [39]. Ferdinand et al. randomized patients to either a placebo or a slowly titrated dose of empagliflozin and observed a blood pressure difference of –8.39 mmHg (95% CI –13.04; *p* = 0.0025) at 24 weeks [40]. A post hoc analysis of the CREDENCE trial revealed a high burden of patients with hypertension; 25% of patients had a systolic BP > 150 mmHg, 30% had resistant hypertension, and 20% were on four or more antihypertensives. This analysis found a reduction of BP of –3.5 mmHg from baseline at week 3, consistent in all hypertensive groups treated with SGLT2i [41]. In all these trials, reductions in blood pressure were seen after several weeks and persisted for at least several months [42]. 

### 4.1. Magnitude of Effect and Patient Selection Considerations

Although these BP improvements are significant, they are small in magnitude, and additional studies are needed to determine their role as primary antihypertensive agents. There are concerns about orthostatic hypotension with the use of any diuretic. However, a meta-analysis of 16 randomized controlled trials (RCT) demonstrated that SGLT2i were not significantly associated with orthostatic hypotension. This meta-analysis noted that if baseline BP ≥ 130/80 mmHg, there was an increasing risk of orthostatic hypotension and a trend toward increased risk in patients with longer duration of T2DM [43].

SGLT2i might have a role in cases of resistant hypertension (RHT, defined as blood pressure above the guideline target despite the use of three or more antihypertensives at optimal doses) [4,44,45]. In a post hoc analysis [46] of the EMPA-REG OUTCOME trial, empagliflozin reduced SBP and DBP by 4.5 mmHg (95% CI 3.1–5.9) and 1.7 mmHg (95% CI 0.9–2.5) in patients with presumed resistant hypertension (pRHT). Although this trial was not designed as an efficacy trial, there was no standard definition of RHT as it did not account for accurate BP measurements, medication adherence, or white coat hypertension.

Lastly, diverse representation in clinical trials is very important, as clinicians must ensure the treatment is effective for other racial/ethnic populations. Black individuals’ participation in these trials is low. Although underrepresentation continues in clinical trials, several efforts have been made to decrease the gap, as these medications can reduce and theoretically eliminate disparities if they are widely prescribed [47,48].

### 4.2. Other Considerations: Safety of SGLT2i

In general, SGLT2i are well-tolerated, and data about their safety has been replicated multiple times. The risk of hypoglycemia appears to be non-significant; however, it is advised to adjust insulin or sulfonylureas if needed and continue blood glucose monitoring [49]. Five large trials (EMPA-REG OUTCOME, DECLARE-TIMI, DAPA-CKD, CANVAS, CREDENCE) did not demonstrate a higher incidence of urinary tract infections (UTIs). Although one meta-analysis showed a higher incidence of UTIs in the SGLT2i group, two others did not show the same findings. Post-marketing data, meta-analysis of RCTs, and observational RCTs have demonstrated an increased risk of genital infections when compared to placebo. Still, most of these events are mild and easily treatable [50]. In severe cases, it is recommended to discontinue SGLT2i. 

Euglycemic diabetic ketoacidosis (DKA) has a low incidence and has been shown to be statistically non-significant. 

Although it is recommended to be cautious about starting SGLT2is in diabetic patients with peripheral vascular disease, only one trial demonstrated a higher risk of lower limb amputation with canagliflozin [31]. However, other major trials did not demonstrate similar results [30,49,51].

## 5. Combining SGLT2 and Non-Steroidal MRAs 

The ROTATE 3 study, a randomized crossover clinical trial of dapagliflozin and eplerenone taken together, demonstrated a presumed physiologic principal that glycosuria may increase potassium excretion, as the combination in the study decreased the risk of hyperkalemia (K serum change from baseline, 0.23 mmol/L) [52]. In a rat model of hypertension, a cardiovascular benefit was demonstrated over the course of 7 weeks with either finerenone, empagliflozin, or a combination of both. There was a 93% survival benefit in the low-dose combination group in comparison to the placebo-treated group (50%) [53]. The combination effect is currently undergoing a phase 2 randomized, controlled, double-blind, double-dummy study, the CONFIDENCE trial (NCT05254002), the primary objective of which is to demonstrate the dual use of empagliflozin and finerenone is superior for reduction of albuminuria. Although the blood pressure effect will be a secondary endpoint, it may shed light on any synergism the combination may have. 

## 6. Guidelines on SGLT2 and Non-Steroidal MRAs on Hypertension Management

In the most recent guidelines for hypertension, SGLT2i, and non-steroidal MRAs are mentioned as having mild to moderate BP effects in comparison to classical anti-hypertensives but with the added benefit of improving cardiovascular and kidney outcomes. The 2023 European Society of Hypertension, in addition to the standard recommendation of starting with ACEi/ARBs, suggests adding SGLT2i for patients with diabetic and non-diabetic CKD if eGFR is at least 20 mL/min/1.73 m^2^. The non-steroidal MRA finerenone is recommended in patients with CKD and albuminuria if eGFR is at least 25 mL/min/1.73 m^2^ as a class I recommendation with an “A” level of evidence [54]. The AHA/ACC/HFSA guidelines for the management of heart failure recommend SGLT2i (2A). However, it is acknowledged that there is a gap in evidence to recommend non-steroidal MRAs for the treatment of heart failure [55].

In the ADA-KDIGO joint statement for the management of diabetes in patients with CKD, SGLT2i is recommended as a first-line add-on therapy, and non-steroidal MRAs are recommended as additional risk-based therapy in patients with persistent albuminuria despite maximum dose of RAAS blockade [56]. Not all patients will be able to tolerate SGLT2i in addition to ACEi or ARB. These patients should be considered for non-steroidal MRA addition if they meet indication and lab qualifications.

## 7. Novel Therapies

It is an exciting time for those who care for patients with diabetic kidney disease. Several agents are being tested for efficacy in this population for additional benefit beyond hypertension control. 

SGLT2i have been increasingly studied on the secondary downstream effects. Some evidence has suggested that chronic hypoxia may be the primary pathophysiological pathway driving diabetic kidney disease and CKD, among other causes. Diabetes mellitus is thought to compromise the oxygen balance by impairing oxygen delivery owing to hyperglycemia-associated microvascular damage and exacerbating oxygen demand owing to increased sodium reabsorption as a result of SGLT upregulation and glomerular hyperfiltration. Adding an SGLT2i may decrease hypoxia-inducible factor-1alpha (HIF-1alpha) in the kidney, revealing less tissue hypoxia [57]. More research is ongoing as we learn more about kidney hypoxia and augmentation.

Pentoxifylline is a nonspecific phosphodiesterase inhibitor that has anti-inflammatory properties. In vitro, it showed promise as it reduced proliferation and differentiation of renal fibroblasts [58]. Subsequently, many RCTs have shown a reduction in albuminuria and a decrease in the slope of eGFR with the use of pentoxifylline in diabetic kidney disease, culminating in the 2019 published PREDIAN study, which demonstrated an impressive 15% change in urinary albumin excretion as well as a significantly decreased rate of eGFR decline when pentoxifylline was added on to RAS blockade in diabetic patients [59]. The large, randomized controlled VA PTXRx trial is underway to provide evidence for the reduction of clinical endpoints such as ESKD and death. 

Meanwhile, while GLP-1 agonists have gained popularity for their role in weight loss and glucose control, their independent renoprotective effects have also come into the limelight. The GLP-1 receptor is expressed in the renal blood vessels, cortex, and tubules, and its inhibition has been shown in vivo and in animal models to decrease pro-fibrotic signaling. For example, in the rat model of obesity-related glomerulonephropathy, TNF-alpha signaling was decreased in response to liraglutide treatment, and morphological changes to podocytes were improved [60]. To that end, the FLOW trial is a randomized controlled trial comparing semaglutide to placebo in patients with proteinuric diabetic kidney disease who are on RAS blockade. The endpoint is a composite of time to kidney failure, eGFR reduction, and renal or cardiovascular death, and the trial is expected to be completed in 2024 [61].

Other novel therapies are upcoming in the kidney space for CKD that may offer unknown insights and clinical improvements. Tirzepatide (LY3298176), a novel insulinotropic polypeptide and GLP-1 combination injection is being studied in combination with ACEi/ARB in the TREASURE-CKD trial (Clinicaltrials.gov: NCT05536804). This study will look at patients with diabetes and outcomes of kidney oxygenation, UACR change, and body weight. Improvements in these parameters may have a positive impact on measured blood pressure concomitantly. The ZENITH-CKD trial (ClinicalTrials.gov: NCT04724837) is ongoing, which combines SGLT2i dapagliflozin with ETA receptor antagonist zibotentan in patients with CKD [62]. Primary outcomes will be UACR change and secondary eGFR slope. Again, blood pressure will be closely monitored, given both agents are linked to lowering blood pressure. Ocedurenone (KBP-5074), a novel non-steroidal MRA, is being studied in the Clarion-CKD trial (ClinicalTrials.gov: NCT04968184) in patients with CKD and uncontrolled hypertension. Primary outcomes will be changes in systolic blood pressure with secondary measures of UACR changes over several months. Adding another MRA to the group may prove to be another tool for this challenging group of patients. Table 1 includes promising ongoing clinical trials for hypertension medication goals in patients with DKD.

## 8. Conclusions

When considering the treatment of hypertension in diabetic patients with chronic kidney disease, a focus should be made to maximize therapy, which demonstrates additional benefits in proteinuria reduction, prevention of eGFR decrease, or cardiovascular benefit. Though hyperkalemia and cost can be dose- or class-limiting, these medications should be maximized when possible. Novel therapies such as pentoxifylline and GLP-1 agonists may provide some blood pressure-neutral interventions for diabetic kidney disease and could be used simultaneously with existing therapies. 

Chronic hypertension is a major public health problem, and existing drugs like SGLT2i and non-steroidal MRAs have shown cardiovascular and kidney benefits in major clinical trials. The addition of SGLT2 inhibitors and finerenone should be considered in the treatment of hypertension as well as resistant hypertension. 

## Figures and Tables

**Table 1:** Ongoing major clinical trials in DKD from ClinicalTrials.gov.

Study Title	Trial Identification	Enrolled	Primary Outcomes
Pentoxifylline in DKD	NCT03625648 PTXRx	2510 patients Pentoxifylline vs. Placebo	Time to ESRD or Death
A Phase 3 Study of Bardoxolone Methyl in Patients with DKD	NCT03550443 AYAME	1323 patients Bardoxolone vs. Placebo	Time to onset of a >30% dec in EGFR from baseline or ESRD
Prognostic Imaging Biomarkers for DKD	NCT03716401 iBEAt	500 patients Biopsy vs. MRI follow-up, Microvascular	Cross-sectional. MRI biomarkers will be combined with fluid-based biomarkers
Comparison Between the Efficacy of SGLT2i vs. ACEi in DKD	NCT05373004 SGLT2i vs. ACEi	212 patients Empagliflozin vs. Enalapril	eGFR rate, UACR measurement
Decision Impact Trial of KidneyIntelX	NCT04791358	1500 patients KidneyIntelX test	Blood pressure, HBA1c, ACEi/ARB, SGLT2i/GLP1, UACR
SGLT2i Prophylaxis Against Post-contrast AKI in DKD	NCT04853615	800 patients Normal Saline vs. Allopurinol/linagliptin vs. SGLT2i vs. SGLT2i/allopurinol	SGLT2i proves protective, non-inferiority to allopurinol
PREvention of CardIovascular and DKD in Type 2 DM	NCT05390892 PRECIDENTD	9000 participants SGLT2i vs. GLP1 vs. SGLT2i/GLP1	Total CV, Kidney, and Death events
Efficacy of a High-Intensity Physical Activity Program on Renal Function	NCT03184662 ACTIDIANE	300 patients Twice weekly activity session vs. Counseling alone	Renal Function Decline
Repository of Novel Analytes Leading to Autoimmune, Inflammatory, and DKD	NCT01802034 RENAL AID	2000 patients Biopsy tissue repository vs. nonuse of RENAL AID	Change in disease progression
Atrasentan in Patients with Proteinuric Glomerular Disease	NCT04573920 AFFINITY	100 patients 0.75 mg atrasentan and 1.5 mg atrasentan (FSGS)	Change in proteinuria, albuminuria (DKD)
Efficacy, Safety, and Tolerability of AZD9977 and Dapagliflozin in Participants with HF and CKD	NCT04595370 MIRACLE	500 patients AZD9977 Dosings + Dapagliflozin	Percent change in UACR at 12 weeks
BI 690517 Alone vs. Combination with Empagliflozin in CKD	NCT05182840	714 patients Empagliflozin + BI 690517 vs. Placebo combinations	Change in UACR
Finerenone and Empagliflozin Combination vs. Alone	NCT05254002 CONFIDENCE	807 patients Finerenone/Empagliflozin, vs. Empagliflozin, vs. Finerenone	Change in UACR at 180 days
Ocedurenone KBP-5074	NCT04968184 Clarion-CKD	600 patients KBP-5074 vs. placebo	Change in SBP at 12 weeks and SBP at 48 weeks

## Data Availability

Not applicable.
